# Early Detection and Longitudinal Follow‐Up of Non‐Invasive Biomarkers for Laryngeal Squamous Cell Carcinoma: First Results of the BEAL‐Study

**DOI:** 10.1002/cam4.70961

**Published:** 2025-05-14

**Authors:** Ann‐Christin Winkelmann, Olaf Wendler, Johannes Schuster, Abbas Agaimy, Marlen Haderlein, Benjamin Frey, Sarina Katrin Mueller

**Affiliations:** ^1^ Department of Otolaryngology, Head and Neck Surgery Universitätsklinikum Erlangen, Friedrich‐Alexander‐Universität Erlangen‐Nürnberg Erlangen Germany; ^2^ Department of Pathology Universitätsklinikum Erlangen, Friedrich‐Alexander‐Universität Erlangen‐Nürnberg Erlangen Germany; ^3^ Department of Radiation Oncology Universitätsklinikum Erlangen, Friedrich‐Alexander‐Universität Erlangen‐Nürnberg Erlangen Germany

**Keywords:** biomarkers, clinical cancer research, head and neck cancer, laryngeal

## Abstract

**Introduction:**

Despite therapeutic and prognostic improvements in the early treatment of laryngeal squamous cell carcinoma (LSCC), screening for early detection of laryngeal cancer has not yet been established.

**Methods:**

A study has been designed to address this issue based on an analysis of exosomal serum biomarkers for LSCC. A three‐step approach was used. Firstly, the serum biomarkers Nov and uPAR were validated in control and LSCC patients using a standardized enzyme‐linked immunosorbent assay (ELISA). Subsequently, an immunohistochemical comparison was conducted. Secondly, the ELISA results were compared with patients with benign laryngeal lesions. Thirdly, a prospective study compared preoperative and postoperative values. The study is ongoing.

**Results:**

In the initial comparison of the control group with tumor patients at the time of diagnosis, Nov and uPAR demonstrated significant overexpression in LSCC patients' serum. In Step 2, the comparison with the cohort of benign laryngeal lesions revealed that both biomarkers were also significantly overexpressed here. Nevertheless, uPAR effectively differentiated between control, benign, and malignant lesions. In the third step, the observations in the postoperative course demonstrated that the serum concentrations of Nov and uPAR were elevated in both groups with laryngeal diseases but declined faster in patients with benign lesions.

**Conclusion:**

uPAR and Nov may serve as valuable biomarkers for early detection and disease monitoring. Further investigation is required to ascertain their suitability, including an extended follow‐up period and a larger study population. For this reason, this study is still ongoing, and only initial results are presented here. This study's objective is to examine the potential of Nov and uPAR as serum biomarkers for early detection and follow‐up of LSCC. We compare them in a control group, tumor group, and first‐time benign laryngeal diseases and monitor a long‐term follow‐up. This aims to deepen understanding of suitability for early detection and follow‐up and provide future research insights.

**Trial Registration:** DRKS00033427

## 1 Introduction

Head and neck tumors represent the fifth most common tumors worldwide [1]. They comprise a heterogeneous group of malignant neoplasms affecting the oral cavity, pharynx, and larynx, which are classified into different tumor entities according to their location [2]. Over 80% of these neoplasms originate from the squamous epithelium [2]. Laryngeal squamous cell carcinoma (LSCC) is one of the most frequent malignant tumors in the head and neck region [3]. In Germany, 4.4 individuals per 100,000 inhabitants are diagnosed with LSCC annually, with a mortality rate of 1.8 per 100,000 [1]. Men are more frequently affected, which is likely attributable to the main risk factors of smoking and alcohol consumption [3]. Despite improved treatment options and a slowly decreasing incidence, mortality rates have remained stable for years [2]. This is mainly due to half of LSCCs being diagnosed at advanced tumor stages, which are associated with a poorer prognosis. Furthermore, as the tumor stage progresses, the therapies become increasingly invasive and can significantly diminish the quality of life for patients. Early diagnosis remains challenging due to unspecific early symptoms, the lack of diagnostic biomarkers, and the absence of established screening programs [3]. At present, patients presenting with hoarseness persisting for more than four weeks or unclear dysphagia undergo an indirect laryngoscopy to ascertain whether there is a suspicious lesion [3]. In the event of a potential malignant lesion being identified, a direct laryngoscopy under anesthesia is performed, which requires hospitalization [3]. A tissue biopsy is obtained and subjected to histopathological examination [3]. However, if the lesion is not situated directly on the vocal folds, the tumor is initially asymptomatic, thereby rendering early detection less probable [3]. A delayed diagnosis is associated with larger tumors, which are linked to a greater loss of functional tissue during treatment [3]. Additionally, there is a higher possibility of systematic spread. Overall, early detection is dependent on the presence of symptoms and is complex and invasive due to the necessity of obtaining biopsies under anesthesia via laryngoscopy.

Liquid biopsies are currently undergoing a significant surge in utilization for the diagnosis of tumors. These samples are primarily blood specimens in which DNA, tumor cells, exosomes, or protein biomarkers can be quantified [4, 5]. As sample collection is non‐invasive, it is ideal for frequent sampling and well‐suited to disease monitoring [5]. The first markers, such as prostate‐specific antigen (PSA), have already been established for prostate cancer monitoring [6]. Given the potential and advantages of liquid biopsies, the objective of our study is to analyze biomarkers from serum.

For the diagnosis and treatment of LSCC, several biomarkers have already been investigated. For instance, the biomarkers cytokeratin 19 fragments (CYFRA 21‐1), tissue polypeptide antigen (TPA‐M), squamous cell carcinoma antigen (SCCA), and carcinoembryonic antigen (CEA) were evaluated in the serum of patients with laryngeal carcinoma and a control group of healthy volunteers [7]. However, none of the markers demonstrated sufficient efficacy for early tumor detection or prediction of tumor behavior during follow‐up [7]. In previous work of our group, we have performed a multiplexed proteomic approach in serum exosomes to identify novel potential biomarkers that are able to discriminate between LSCC and control patients [8]. We could identify nephroblastoma overexpressed protein (Nov) and urokinase‐type plasminogen activator receptor (uPAR) as promising biomarkers.

Nov has been gaining importance and scientific interest in breast tumors, prostate cancer, and other malignancies for several years. Nov, also known as CCN3 or IGFBP‐9, belongs to the CCN protein family, which is named after the first three of the six CCN proteins [9, 10, 11]. These proteins are secreted and share four homologous domains: insulin growth factor‐binding protein, von Willebrand factor type C, thrombospondin type 1 repeat, and carboxy‐terminal knot. Due to their binding sites, CCN proteins act in numerous different processes [9].

Nov can promote cell adhesion, cell migration, cell survival, and angiogenesis by binding to integrins [12, 13]. Lin et al. [12] were able to demonstrate the direct binding of Nov to integrins, in particular integrin α_V_β_3_. This integrin promotes endothelial cell attachment, mitogenic growth factor synthesis, and cell movement [12]. Moreover, Nov has been demonstrated to induce neovascularization via integrin binding in experiments conducted on rat corneas [12]. Additionally, CCN proteins can act intracellularly and influence the cell cycle [14, 15].

The concentration of Nov varies in different tumors, and the functions of the protein in the different entities are also diverse [16]. Increased expression levels can indicate greater differentiation, but also increased proliferation and metastasis rates. In prostate, renal cell carcinomas, and osteosarcomas, overexpression of Nov was associated with increased tumor cell proliferation [16, 17]. In chronic myeloid leukemia and glioblastoma, however, Nov appears to have more anti‐proliferative effects [18, 19]. The protein also has anti‐inflammatory and regenerative effects in autoimmune diseases [20, 21, 22].

The protein uPAR is a membrane‐bound protein receptor located on the outer surface of cells and is part of the uPA/uPAR system, which is involved in a proteolytic cascade [23]. The pro‐urokinase‐type plasminogen activator (pro‐uPA) can be cut to uPA by proteases such as cathepsins [24]. Plasminogen is cleaved by the binding of the ligand uPA to uPAR [23, 25]. The cleaved and thereby activated plasmin initiates remodeling of the extracellular matrix and activates matrix metalloproteases [25]. This increased protease activation favors local cell migration by modifying the actin cytoskeletal structure of cells and the release of growth and angiogenesis factors [26]. Via transmembrane proteins, uPAR can also influence intracellular signaling cascades that increase cell proliferation [27]. By inducing proteolysis and proliferation, uPAR promotes tumor growth, tissue invasion, and metastasis [27]. The transforming growth factor β (TGFβ) activates both uPAR and cathepsins, thereby having an impact on the extracellular matrix remodeling as well [28].

Previous research has shown that uPAR is overexpressed in various tumor tissues, including LSCC, and is associated with poor prognosis [29, 30, 31, 32, 33]. Furthermore, the overexpression of the marker has been confirmed in serum samples obtained from patients diagnosed with different tumors, including those of the head and neck, cervix, breasts, prostate, pancreas, colon, and lungs [34, 35, 36, 37, 38, 39, 40, 41]. In addition to its role in tumorigenesis, uPAR is implicated in inflammatory processes and wound healing [42, 43].

A previous study has identified overexpression of uPAR in invasively obtained tissue samples from LSCC patients [32]. The study by Schmidt and Hoppe [40] on elevated uPAR serum levels in head and neck squamous cell carcinoma (HNSCC) also included a small subgroup of 13 patients with LSCC. Furthermore, Risor et al. [41] examined uPAR in the serum of 5 LSCC patients prior to radiotherapy and after 2 months to compare these results with those obtained from uPAR positron emission tomography/computed tomography (PET/CT). These initial results, generated in small study groups, will be re‐evaluated as part of this study.

The plasminogen activation pathway already represents a promising avenue for therapeutic intervention. Recent findings indicate the involvement of cathepsins as well. Gogineni et al. [28] demonstrated that deactivating uPAR and cathepsin reduces cell migration and invasion.

Whereas Nov and uPAR have been identified as promising novel biomarkers for LSCC, fundamental research is still missing. While a study on uPAR in LSCC patients does present pre‐ and postoperative comparisons, no studies at all have yet been conducted on the potential of Nov as a serum biomarker in laryngeal carcinomas. Furthermore, there is no research examining the biomarker levels in benign laryngeal lesions. Additionally, there is a paucity of studies analyzing biomarker concentrations over extended periods.

To address these gaps in the literature, we will investigate Nov as a biomarker for LSCC in serum for the first time, analyze both biomarkers in benign laryngeal lesions for the first time, and conduct a regular long‐term follow‐up, which has not been done previously. Therefore, the objective of this study was (1) to validate the biomarkers in whole serum for early detection of LSCC, (2) to compare serum biomarker levels for control, benign lesions, and malignant lesions, and (3) to follow‐up serum biomarkers at different time points to evaluate the clinical meaningfulness.

## 2 Methods

### 2.1 Study Design

This was a prospective translational study (ethics committee of the Friedrich‐Alexander‐University Erlangen‐Nuremberg [FAU], No. 451_20 B) of to date 140 patients that were treated at our tertiary care hospital between September 2020 and June 2023. The study design consisted of a three‐step process to enhance the study's intelligibility. The first step was to validate the results of an exosomal multiplex array from the preceding study by Schuster et al. [8]. This was done by comparing the levels of Nov and uPAR in LSCC patients with those of a control group at the time of diagnosis to assess the sensitivity of the biomarkers. Secondly, a comparison was made between the control group and malignant and benign laryngeal diseases to address the markers' specificity. This was the first time a cohort of patients with benign laryngeal conditions was examined. Thirdly, the prospective follow‐up of Nov and uPAR levels over time was observed. Thus far, the study has enrolled a subset of the LSCC cohort from the first step and several patients from the benign group of the second step. As the study progresses, the remaining individuals from the two groups will also participate in the follow‐up and be evaluated. Furthermore, for the first time, postoperative data were included for Nov. The prospective translational part of the study was registered as a prospective observational study (BEAL: Biomarkers for Early detection And Longitudinal follow‐up for LSCC, DRKS00033427). Here, first data are presented. The study is ongoing.

### 2.2 Study Population

Three patient cohorts were included in the study. The first cohort comprised patients who had undergone septorhinoplasty (control group), the second comprised patients with benign laryngeal lesions, and the third comprised patients with LSCC. The preoperative samples of the cohort were employed to corroborate the outcomes of the multiplexed proteomic assay and to contrast the aforementioned groups with one another. From a subset of the cohort, postoperative samples were used to conduct a prospective follow‐up over time.

**FIGURE 1:**
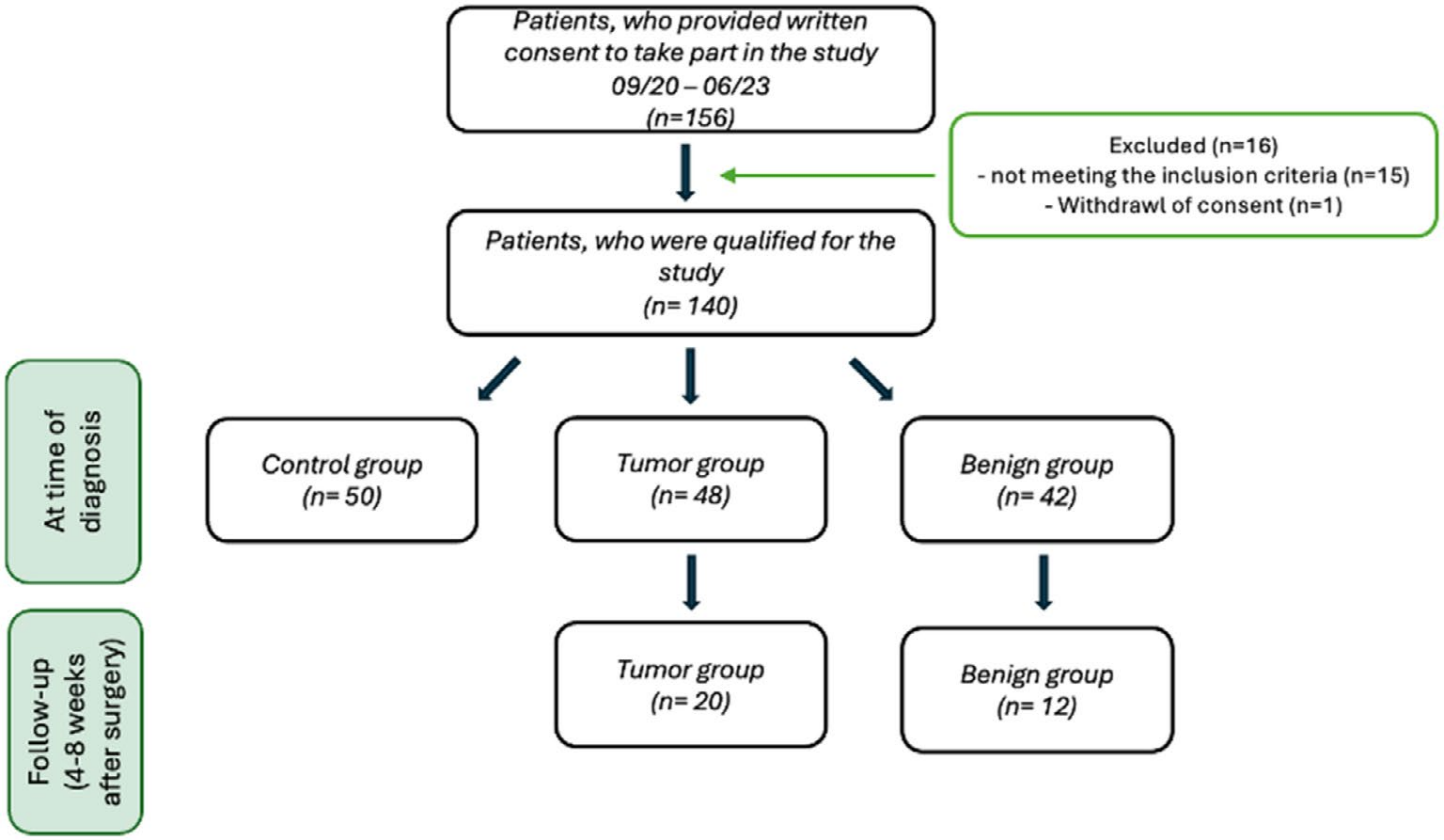
The flow chart provides an overview of the study design employed in this study.

In detail, the study group for Steps 1 and 2 comprised a control group of *n* = 50, a group with benign laryngeal lesions of *n* = 42, and a group with laryngeal tumors of *n* = 48 (see Figure 1). Of this study group, 20 patients with LSCC and 12 participants with benign laryngeal lesions underwent a follow‐up examination for Step 3 (see Figure 1).

Control patients were healthy patients undergoing septorhinoplasty for functional nasal blockage without the history of cancer. Blood was sampled from all patients 1 day prior to surgery at hospital admission between 8 am and 1 pm and processed within 1 h. The benign and malignant laryngeal lesion group also had a tissue biopsy taken during panendoscopy. The panendoscopy was used to determine the extent of the lesion and to sample the biopsy. The biopsy was then sent to the Pathological Department of the Friedrich‐Alexander‐University Erlangen‐Nürnberg. TNM and UICC (Union Internationale Contre le Cancer, 8th version) staging systems were determined after histopathological confirmation and according to computed tomography (CT) imaging [44]. TNM and UICC staging systems were adapted after final tumor therapy if needed.

All participating patients gave written consent to take part in the study and were of legal age. The inclusion criteria for the tumor group consisted of patients with a newly occurring squamous cell tumor of the larynx that was histologically confirmed and not treated before surgery. Exclusion criteria were patients with other malignant tumors in the last 5 years, distant metastases, chronic inflammatory diseases, and autoimmune diseases. The use of immunosuppressants also led to exclusion. For the benign laryngeal group, patients with leukoplakia, vocal fold polyp, contact granuloma, irritative granuloma, or laryngocele were included. As with the tumor group, patients with malignant diseases within the last 7 years, chronic inflammatory conditions, autoimmune diseases, and those taking immunosuppressants were excluded from the benign group. The control group comprised patients who had received septorhinoplasty due to an acquired nasal deformity and who were of a similar age to the tumor group. Patients with malignant diseases, autoimmune diseases, septorhinoplasty revisions, and patients who underwent surgery to correct functional nasal pathologies such as nasal obstruction, obstructive sleep apnea syndrome (OSAS), or inflammatory changes or were taking immunosuppressants were excluded from the study. The aim was to ensure that the patient cohort was as healthy as possible, thereby providing a suitable control group.

To address potential confounding factors, care was taken to ensure that the patients in the different study groups had similar demographic characteristics, including age, gender, and co‐morbidities. Furthermore, the inclusion and exclusion criteria were designed to minimize the influence of other tumor diseases and autoimmune diseases, as well as certain medications. By taking blood samples 1 day before the operation, it can be assumed that the patients did not have a current infection, as otherwise they would not have been operated on. This potential confounding factor was therefore also addressed. After the surgical procedure, an interval of 4 weeks was observed until the next blood sample was taken in order to minimize the impact of potential alterations in blood values resulting from the manipulation of the larynx.

The BEAL study was approved by the Ethics Committee of the Friedrich‐Alexander University Erlangen‐Nuremberg (No. 20‐451‐B). All study procedures were conducted according to the standards of the Ethics Committee and the Declaration of Helsinki. In compliance with the ethical requirements pertaining to the confidentiality of patients, all patient data were pseudonymized prior to analysis.

### 2.3 Follow‐Up

Blood was taken preoperatively as well as after a follow‐up scheme based on the routine follow‐up regimen of all head and neck tumor patients. This ensures that samples can be collected at regular, fixed intervals over an extended period without imposing an additional burden on the patient. Furthermore, the patient's current clinical condition can be evaluated concurrently with the sampling process, and potential confounding factors, such as current infections that may impact biomarker levels, can be minimized. The follow‐up interval after surgery was every 4–8 weeks during the first year, every 3 months during the second and third years, and every 6 months beginning in the fourth year. At these time points, blood samples were collected. In this preliminary data, the first pilot results of the first postoperative time point will be presented. The Step 3 trial group currently includes 20 patients with LSCC and 12 participants with benign laryngeal lesions. The patients' follow‐up appointments were scheduled between 8 am and 1 pm. Blood samples were also collected at this time. The blood was processed in our laboratory within an hour. The preoperative biomarker levels were set as baseline for the postoperative follow‐up levels.

### 2.4 Serum Preparation

The whole blood was collected in a serum tube from 8 am to 1 pm and then left to coagulate for 30 min at room temperature. The serum was then centrifuged at 3000×*g* for 10 min, aliquoted into four 0.5 mL tubes, and stored at −80°C until analyzed.

### 2.5 Analysis of Serum by Enzyme‐Linked Immunosorbent Assay (ELISA)

The protein concentrations of the biomarkers Nov and uPAR were determined in the patient serum samples using ELISA. A total of 48 patients with laryngeal carcinoma, 42 patients with benign laryngeal lesions, and 50 control group probands (septorhinoplasty) were analyzed. The assays were performed using the DuoSet human Nov/CCN3 and human uPAR ELISA kits (DY1640, DY807 R&D Systems Inc., Minneapolis, MN).

Nov was prediluted 1:20 with Reagent Diluent (1% Bovine serum albumin in PBS phosphate buffered saline), while a predilution of 1:15 with the same reagent was used for uPAR.

The ELISAs were carried out in strict compliance with the manufacturer's protocols. The standard curves and the results were recorded (Multiskan Go, Thermo Fisher Scientific, Bonn, Germany) and calculated using the SkanIt software 4.1 for microplate readers (Thermo Fisher Scientific, Bonn, Germany). In order to ensure the standards of quality control, the standard series and the samples were measured in triplicate, and two internal quality standards were also included in each measurement. Furthermore, the Multiskan reader used is calibrated on an annual basis with a spectrophotometric verification plate.

### 2.6 Tissue Analysis by Immunohistochemistry (IHC)

Immunohistochemical staining was performed to visualize the biomarkers in laryngeal tissue. The LSCC tissue obtained during surgery was promptly cut and preserved in 4% formalin for a period of up to 24 h, contingent on the dimensions of the tissue sample. The tissue specimens were subsequently conveyed to the pathology department, where they underwent a process of dehydration and embedding. The tissue sections were then cut into thin slices, stained, and analyzed by the pathologists. Some unstained slides were dispatched to our laboratory for immunohistochemical staining. Normal tissue sections were prepared from healthy laryngeal tissue for comparison with the tumor tissue.

The immunohistochemistry of Nov and uPAR was performed using a ZytochemPlus AP Polymer‐Kit (Zytomed Systems, Berlin, Germany). The tissue sections were deparaffinized and rehydrated. Antigen unmasking was achieved by treatment with a 10 mM citrate‐unmasking agent at 95°C for 20 min. This was followed by incubation with 0.1% Triton X‐100 at room temperature for 5 min. To minimize background staining, the sections were coated with BLOXALL (Vector Laboratories, Newark, US) for 10 min, followed by a protein block using the kit's block solution for 5 min. The antibodies Nov (Clone D‐9) and uPAR (Clone E‐3, Santa Cruz Biotechnology, Heidelberg, Germany) were diluted 1:40 each and applied to label the antigens of interest. The prepared sections were incubated overnight at 4°C in a humidity chamber. Negative controls were obtained by adding a non‐specific mouse antibody (Cell Signaling Technology Inc., Danvers, MA). This was followed by application of the PostBlock and AP Polymer reagent from the ZytochemPlus AP Polymer‐Kit in use. Subsequently, the samples were incubated in SIGMAFAST Fast Red TR/Naphthol AS‐MX (Sigma‐Aldrich, Taufkirchen, Germany) for 10 min, followed by counterstaining with Harris' hematoxylin solution (ORSAtec GmbH, Bobingen, Germany). The slides were then covered with Aquatex (Merck, Darmstadt, Germany) and photographs of the specimens were taken using the BZ‐X810 microscope and the corresponding software (Keyence, Neu‐Isenburg, Germany).

A semi‐quantitative analysis of the immunohistochemical staining was performed, with a rough distinction made according to staining intensity.

### 2.7 Statistical Analysis

A two‐tailed unpaired *t*‐test was conducted to compare the control group with the benign group and with the different tumor group subdivisions (T1, T2, T3‐4, T1‐4) prior to surgery. The same test was employed to compare the results of the benign group pre‐ and postoperatively in a general sense, without analyzing the individual courses. Furthermore, an unpaired *t*‐test was utilized for a comparison of serum levels of LSCC patients before and after surgery. Additionally, the test was conducted to evaluate the postoperative outcomes of the tumor group in comparison to the benign group. A paired two‐tailed *t*‐test was performed to assess the serum levels of Nov and uPAR before and after surgery in the benign and tumor group, considering the individual course of each patient. Every *t*‐test was analyzed using GraphPad Prism software (GraphPad Prism 10.2.0; GraphPad Software, La Jolla, CA). All tests were conducted with an alpha error of 0.05. Prior to this, all data was analyzed for outliers, and a normal distribution test was performed with the GraphPad Prism software. A value of *p* < 0.05 was considered statistically significant to detect differences between the groups analyzed. A multivariate binary logistic regression was employed to investigate the impact of age, gender, and smoking status on Nov and uPAR, as well as to test the diagnostic accuracy and predictive capacity of Nov and uPAR individually and in combination. Therefore, three model variants were created, which included the analysis of the control and tumor group, the control and benign group, and the benign and tumor group. The analysis was done utilizing the statistical software package SPSS (IBM SPSS Statistics, version 19.0.2.0; International Business Machines Corp., Armonk, NY). Similarly to the aforementioned analyses, an alpha error of 0.05 was employed, with *p* values < 0.05 deemed to be statistically significant.

## 3 Results

The demographics of the study populations are displayed in Table 1.

**TABLE 1:** Demographic data for all tumor patients, benign group and control patients.

Characteristics in (%)	preOP				Follow up care (first follow‐up time point)		
	Control (*n* = 50)	Tumor group (*n* = 48)	Benign group (*n* = 42)	*p*	Tumor group (*n* = 20)	Benign group (*n* = 12)	*p*
Age (years), mean ± SD	31.6 ± 12.2	65.1 ± 8.28	52.2 ± 14.3	*p* < 0.0001	65.5 ± 7.46	50.4 ± 11.5	*p* < 0.0001
Gender							
Male	30/50 (60.0)	44/48 (91.7)	24/42 (57.1)	*p* = 0.0001	19/20 (95.0)	7/12 (58.3)	*p* = 0.0185
Female	20/50 (40.0)	4/48 (8.33)	18/42 (42.9)	*p* = 0.0001	1/20 (5.00)	5/12 (41.7)	*p* = 0.0185
Causasian	50/50 (100)	48/48 (100)	42/42 (100)	*p* > 0.9999	20/20 (100)	12/12 (100)	*p* > 0.9999
Tumor size (T)							
T1	—	21/48 (43.8)	—		13/20 (65.0)	—	
T2	—	7/48 (14.6)	—		3/20 (15.0)	—	
T3	—	7/48 (14.6)	—		2/20 (10.0)	—	
T4	—	13/48 (27.1)	—		2/20 (10.0)	—	
Nodal status (N)							
N0	—	30/48 (62.5)	—		18/20 (90.0)	—	
N1	—	4/48 (8.33)	—		0/20 (90.0)	—	
N2	—	8/48 (16.7)	—		0/20 (0.00)	—	
N3	—	6/48 (12.5)	—		2/20 (10.0)	—	
Distant metastasis (M)							
M0	—	47/48 (97.9)	—		20/20 (100)	—	
M+	—	1/48 (2.08)	—		0/20 (0.00)	—	
Benign laryngeal lesions							
Leukoplakia	—	—	12/42 (28.6)		—	3/12 (25.0)	
Vocal fold polyp	—	—	25/42 (59.5)		—	9/12 (75.0)	
Contact granuloma	—	—	1/42 (2.38)		—	—	
Irritative fibroma	—	—	1/42 (2.38)		—	—	
Laryngocele	—	—	1/42 (2.38)		—	—	
Comorbidity							
Asthma	1/50 (2.00)	7/48 (14.6)	8/42 (19.0)	*p* = 0.0154	3/20 (15.0)	0/12 (0.00)	*p* = 0.2742
Adipositas	3/50 (6.00)	14/48 (29.2)	15/42 (35.7)	*p* = 0.0006	6/20 (30.0)	6/12 (50.0)	*p* = 0.2883
Arterial hypertension	1/50 (2.00)	35/48 (72.9)	17/42 (40.5)	*p* < 0.0001	15/20 (75.0)	5/12 (41.7)	*p* = 0.2499
(former) Smoker	18/50 (36.0)	40/48 (83.3)	25/42 (59.5)	*p* < 0.0001	15/20 (75.0)	4/12 (33.3)	*p* = 0.0300
Occasional/moderate alcohol consumption	28/50 (56.0)	23/48 (47.9)	18/42 (42.9)	*p* = 0.429	8/20 (40.0)	5/12 (41.7)	*p* > 0.9999

The control group exhibited a significantly lower prevalence of comorbidities, including asthma, obesity, and arterial hypertension, as well as a lower rate of alcohol consumption and a smaller number of smokers compared to the benign and LSCC groups. The benign and tumor groups showed no significant differences in the previously mentioned characteristics before surgery.

### 3.1 Serum Nov Is Significantly Overexpressed in LSCC and Benign Laryngeal Lesions Compared to Controls

**FIGURE 2:**
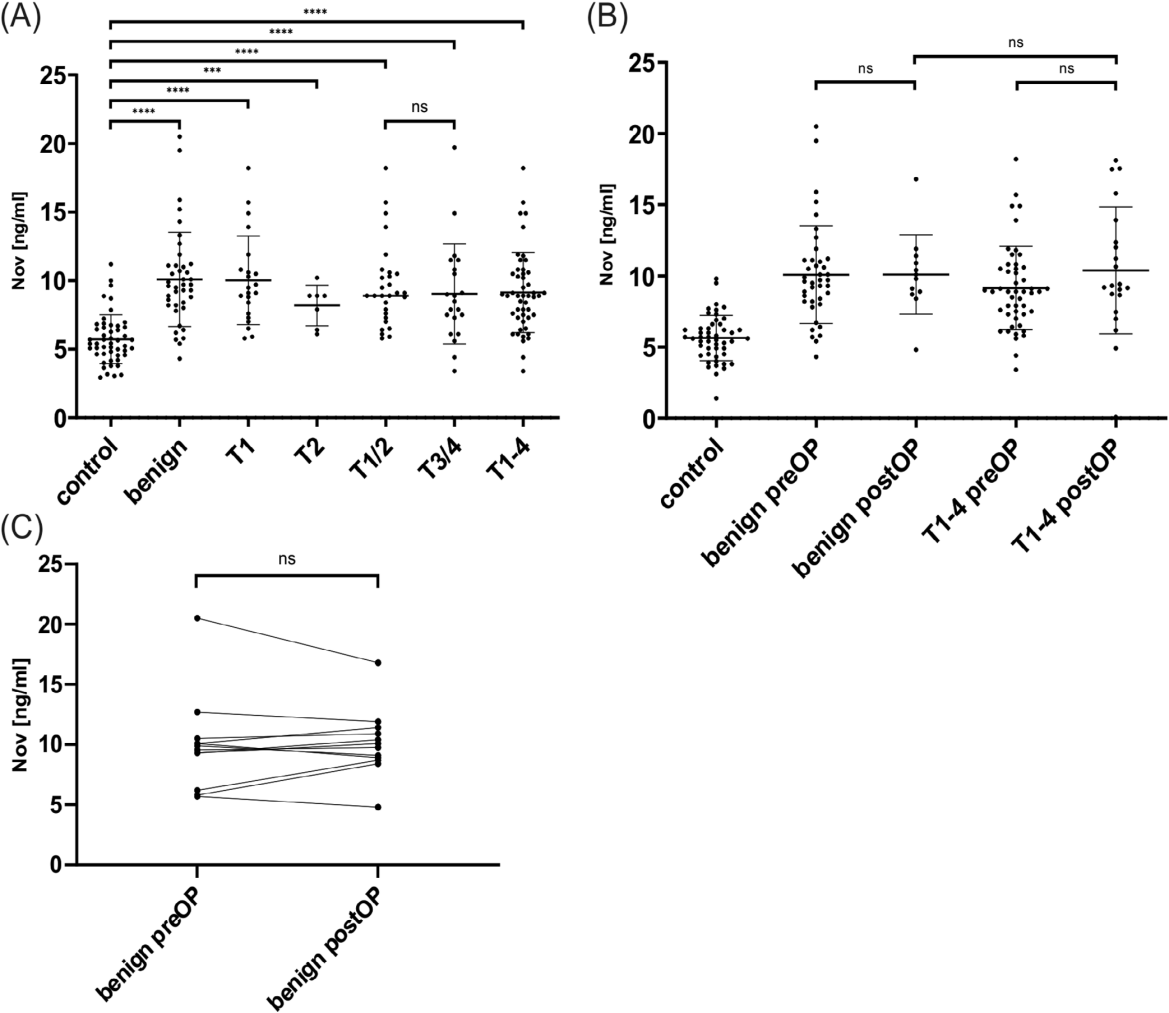
The ELISA results of the comparative analysis of Nov levels between controls, benign laryngeal lesions and laryngeal squamous cell carcinoma (LSCC). (A) Prior to surgery, there was a significant overexpression in both the benign and tumor groups compared to the control group. (B) However, no differences were found when comparing pre and postoperative samples (1–3 months after surgery) within the benign and tumor groups. (C) The presentation of individual patients with benign lesions thin the benign and tumor groups before and after surgery serves to illustrate the course of Nov serum levels. ns = not significant, ***p < 0.001, ****p < 0.0001.

In order to validate the results of our multiplexed proteomic approach, we first performed a validation of the promising proteins Nov and uPAR using ELISA. Preoperatively, Nov levels in the different study groups were compared between LSCC and control patients. This revealed a significant overexpression of Nov in LSCC patients compared to the control group (control vs. T1‐4, *p* < 0.0001, see Figure 2A). A breakdown of the tumor groups by TNM classification also showed a significant overexpression in each TNM class compared to the control group (control vs. T1, T1/2, T3/4, *p* < 0.0001; control vs. T2, *p* < 0.001, see Figure 2A). Therefore, it can be concluded that both the low and high TNM classes of LSCC can be distinguished from the controls. A breakdown of the data by LSCC localization into supraglottic (mean serum level of 9.820 ng/mL), glottic (mean serum level of 9.395 ng/mL) and a group with at least two affected localizations (mean serum level of 8.153 ng/mL) revealed no statistically significant differences. The comparison between the control group and the group of patients with benign laryngeal changes showed a significant overexpression of Nov levels in the latter group (control vs. benign group, *p* < 0.0001, see Figure 2A). However, the biomarker was not able to discriminate between the benign laryngeal lesions and the tumor groups, especially T1. Further detailed information, including mean differences and confidence intervals, can be found in Table 2.

**TABLE 2:** The mean differences and associated confidence intervals between the analyzed patient groups.

	Nov (ng/mL)	uPAR (ng/mL)
Control vs. benign	4.36 [3.24 to 5.47]	4.91. [4.17 to 5.65]
Control vs. T1	4.29 [3.11 to 5.47]	3.13 [2.47 to 3.79]
Control vs. T2	2.46 [1.04 to 3.88]	3.46 [2.67 to 4.24]
Control vs. T1/2	3.85 [2.78 to 4.92]	3.21 [2.58 to 3.84]
Control vs. T3/4	3.32 [2.03 to 4.60]	4.48 [3.64 to 5.32]
Control vs. T1‐4	3.41 [2.45 to 4.38]	3.59 [2.95 to 4.23]
T1/2 vs. T3/4	−0.53 [−2.42 to 1.35]	1.27 [−0.09 to 2.63]
Benign preOP vs. postOP	0.02 [−2.17 to −2.20]	−3.75 [−5.20 to −2.22]
T1‐4 preOP vs. postOP	1.25 [−0.55 to 3.04]	0.18 [−0.98 to 1.34]

### 3.2 Immunohistochemical Staining Confirms Nov Overexpression in LSCC Tissue

**FIGURE 3:**
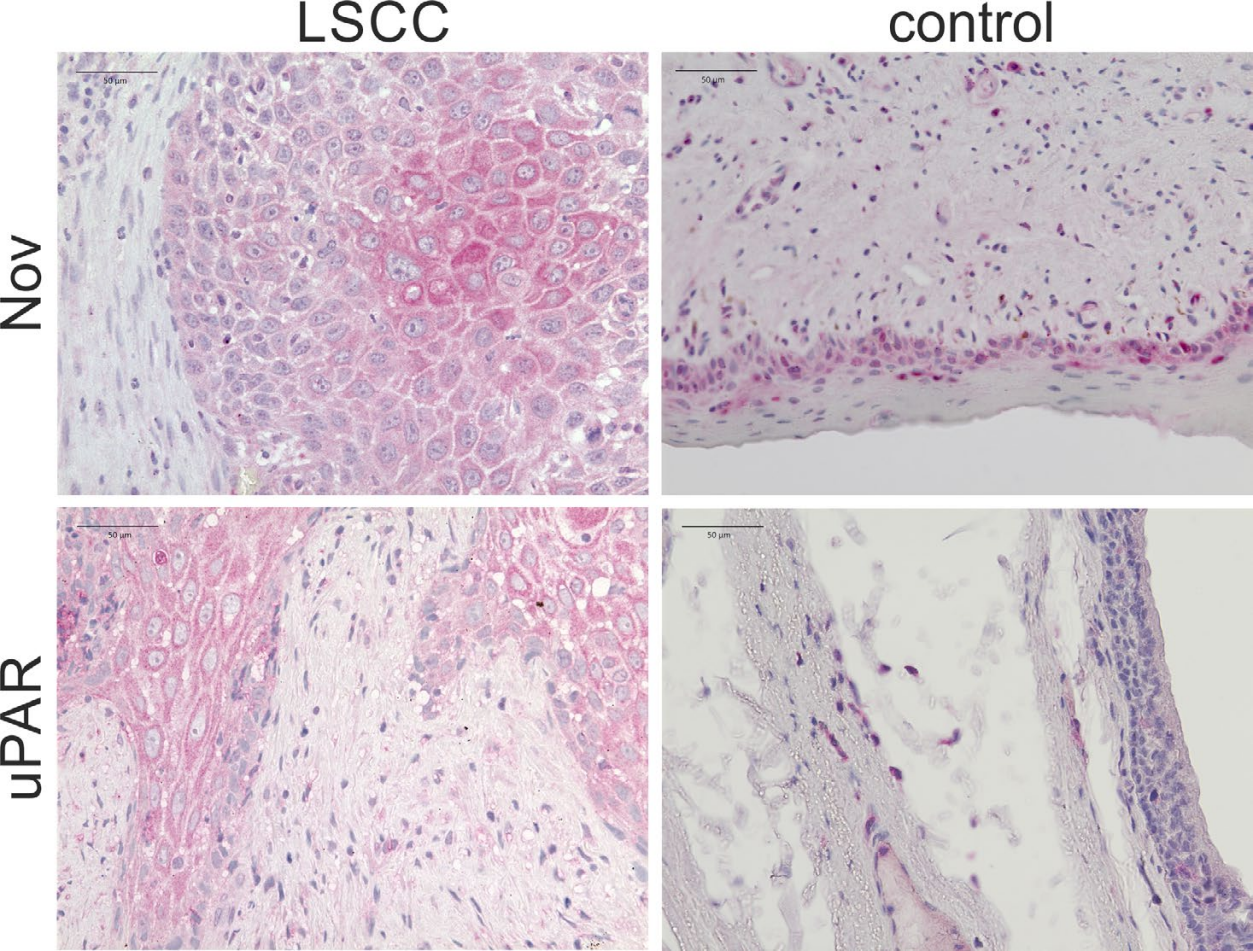
The immunohistochemical staining of the biomarkers Nov and uPAR in laryngeal squamous cell carcinoma (LSCC) samples compared to control tissue indicates a significant overexpression of both markers in epithelial tumor tissue. A 50 μm scale is provided in the upper left corner of each image.

The immunohistochemical staining of Nov is consistent with the results obtained from the ELISA. A notable overexpression of Nov has been observed in LSCC tissue excised from the core of the primary tumor. The epithelial tumor cells have strongly stained cytoplasm, with the center exhibiting an even more intense staining. The surrounding stromal cells are almost unstained. Only the fibroblast‐like cells of the stroma appear to be faintly immunostained. In non‐tumor laryngeal tissue obtained from the periphery of the biopsied tissue, only a slight staining of the basal squamous epithelial layers is visible, which does not persist in the more superficial cell layers. The staining does not extend beyond the basement membrane. The underlying layers are unstained (see Figure 3).

### 3.3 Significant Decrease in Serum Nov Levels at the First Follow‐Up Time Point

The first follow‐up time point of the prospective observational study was performed in a subset of the cohort. Nov levels in LSCC patients and benign lesions were significantly overexpressed at this time point. However, Nov levels in LSCC did not change significantly in comparison to the preoperative time point. In benign lesions, there was a trend towards a decrease in the postoperative period. A comparison of postoperative serum levels of Nov between the benign and the tumor group revealed no significant difference at the first follow‐up point (see Figure 2B). The individual courses of patients in the benign group also demonstrated no observable decline in Nov concentration following surgery (see Figure 2C). Further analysis details may be found in Table 2.

### 3.4 Significant Overexpression of Serum uPAR Levels in LSCC as Well as Benign Laryngeal Lesions Compared to Controls

**FIGURE 4:**
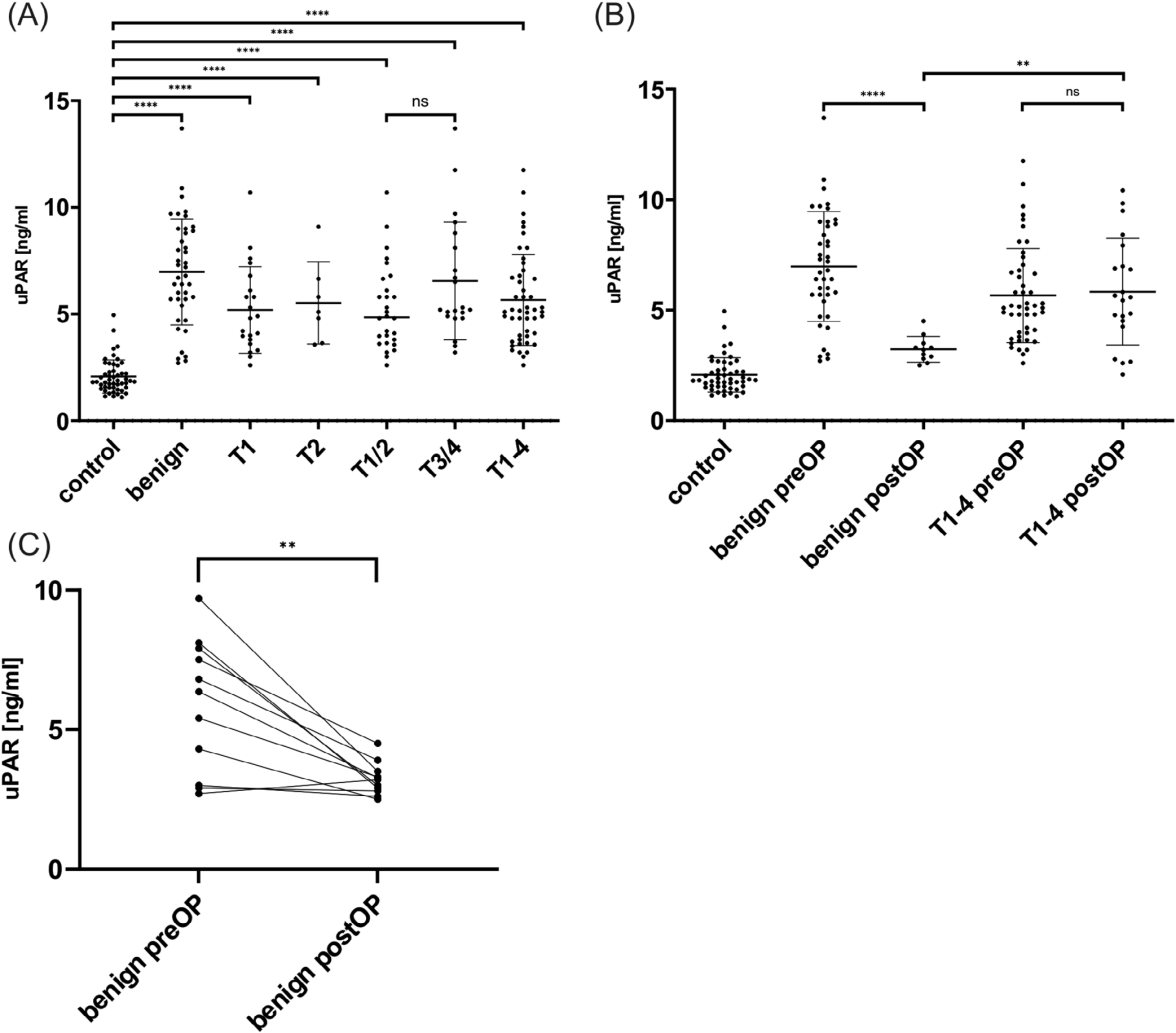
The ELISA results of the comparative analysis of uPAR levels between controls, benign laryngeal lesions and laryngeal squamous cell carcinoma (LSCC). (A) Preoperatively, both benign and tumor groups exhibited significant overexpression compared to the control group. (B) In the follow‐up period of 1–3 months, the uPAR concentration in the benign group decreased significantly compared to the preoperative state, while no changes were observed in the tumor group. (C) The presentation of individual patients with benign lesions before and after surgery underlines the decrease in uPAR serum levels after surgery. ns = not significant, **p < 0.01, ***p < 0.001, ****p < 0.0001.

The protein uPAR was investigated as a second promising biomarker. Here again, uPAR was significantly overexpressed in the LSCC group compared to the control group before surgery (control vs. T1‐4, *p* < 0.0001, see Figure 4A). The subdivision of patients according to tumor location into supraglottic (mean serum level uPAR 6.395 ng/mL), glottic (mean serum level uPAR 5.437 ng/mL) and a group with at least two affected locations (mean serum level uPAR 5.402 ng/mL) yielded no statistically significant results. A breakdown of the tumor groups by TNM classification reveals a significant overexpression in the early and advanced tumor classes when compared to the control group (control vs. T1, T2, T1/2, T3/4, *p* < 0.0001, see Figure 4A). Nevertheless, no notable differences in uPAR levels were observed between early and advanced tumors. But uPAR could successfully distinguish between the control group and the group of benign laryngeal lesions (control vs. benign group, *p* < 0.0001, see Figure 4A). More detailed information on mean differences and confidence intervals can be found in Table 2.

### 3.5 Immunohistochemical Staining Confirms Overexpression of uPAR in LSCC Tissue

The immunohistochemical imaging of uPAR in LSCC tissue reveals strong cytoplasmic staining of malignant epithelial cells, thereby confirming the overexpression of uPAR in the cells of the tumor core. The dysplastically altered cell nuclei of the squamous epithelium and the intercellular spaces remain unstained. The stromal tissue exhibits only weak staining, which appears to be assignable to fibroblast‐like cells. In the laryngeal tissue of the control group obtained from the periphery of the biopsies, uPAR is not detectable in the epithelium. The isolated stainings observed below the epithelium are most probably connective tissue cells (see Figure 3).

### 3.6 T1 Tumors and Benign Laryngeal Lesions Can Be Differentiated by uPAR Serum Levels at Baseline

uPAR levels were overexpressed in LSCC and benign lesions compared to controls at baseline, as described above. Further, uPAR could also differentiate between benign lesions and LSCC (benign group vs. T1‐4, *p* = 0.0085, see Figure 4A). Interestingly, uPAR was able to distinguish between benign laryngeal lesions and T1 tumors preoperatively (benign group vs. T1, *p* = 0.0063; see Figure 4A).

### 3.7 Significant Decrease in Serum uPAR Levels at the First Follow‐Up Time Point

Looking at the uPAR serum levels at the first follow‐up time point, it can be noted that there was a decrease in the benign lesion group (preOP vs. postOP, *p* < 0.0001, see Figure 4C). Although it was a drastic reduction, no significance could be noted due to the low sample size. Interestingly, here again, uPAR levels in LSCC patients did not decrease that steeply and remained rather stable at the first follow‐up time point (benign postOP vs. T1‐4 postOP, *p* = 0.0015, see Figure 4B). Further detailed information regarding mean differences and confidence intervals can be found in Table 2.

### 3.8 Control and Laryngeal Lesions Can Be Well Differentiated by Combing Nov and uPAR

**FIGURE 5:**
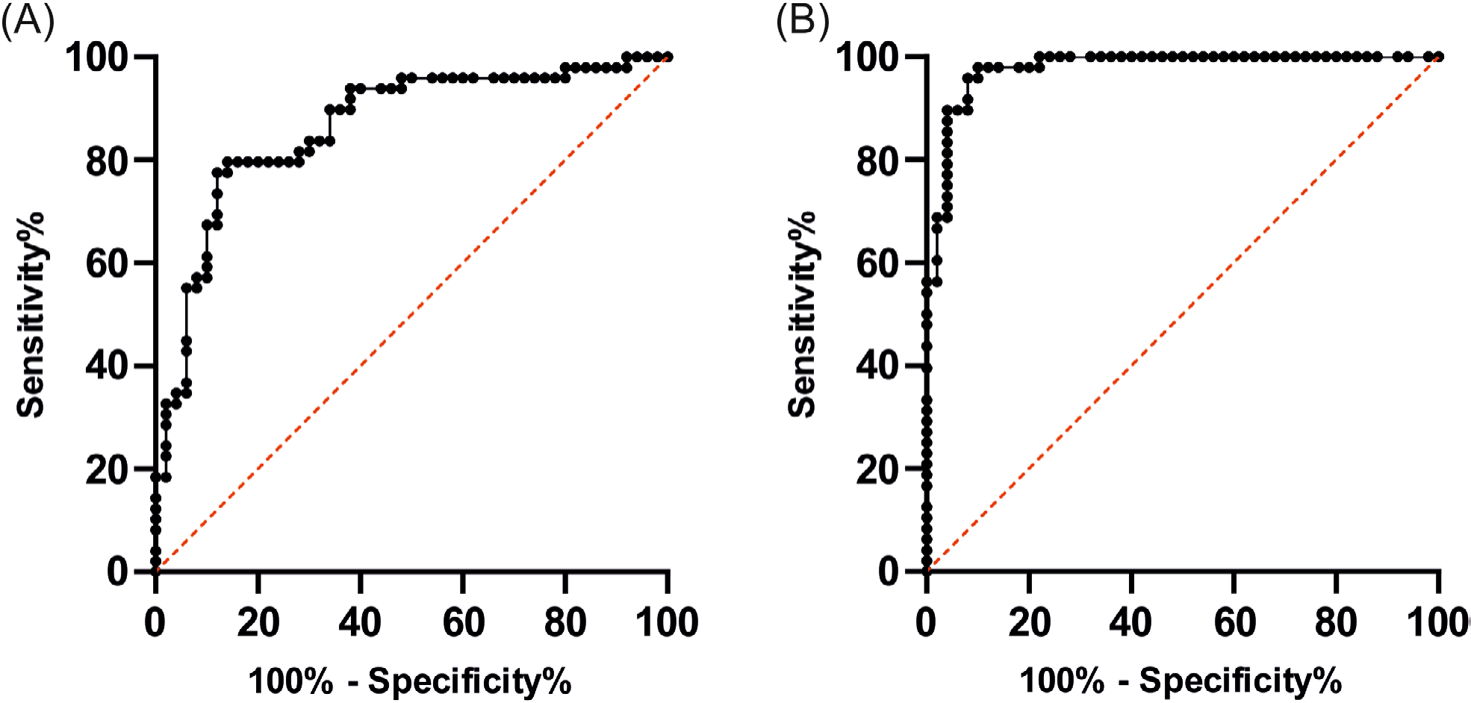
(A) The receiver operating chrarcteristic (ROC) curve illustrates the results for Nov. The area under the curve is 0.8641, with a cut‐off value set at a sensitivity of 0.796 and a specificity of 0.86. (B) The ROC curve shows the biomarker uPAR. The area under the curve is 0.9775, with a cut‐off value of uPAR exhibiting a sensitivity of 0.958 and a specificity of 0.92.

Firstly, a receiver operating characteristic (ROC) curve was employed for a more precise evaluation of the discriminatory power of Nov and uPAR separately. The analysis of the control and tumor groups for Nov yielded an area under the curve of 0.8641 (see Figure 5). The optimal cut‐off value was determined to be a sensitivity of 0.796 and a specificity of 0.860 (see Figure 5). The uPAR testing revealed an area under the curve of 0.9775 for uPAR (see Figure 5). The optimal cut‐off value was determined to be 0.958 for sensitivity and 0.920 for specificity (see Figure 5).

A binary logistic regression was conducted to ascertain the predictive capacity of Nov and uPAR in conjunction with one another. The model comprising the control and tumor groups yielded statistically significant results. The model demonstrated a sensitivity of 0.917 and a specificity of 0.913 in predicting the diagnosis. The overall accuracy was 0.915. Furthermore, uPAR demonstrated a statistically significant impact on the diagnosis (*p* < 0.001). The same was true for the analysis conducted with the control and benign groups. The test quality was characterized by a sensitivity of 0.902, a specificity of 0.957, and an accuracy of 0.931. In addition, Nov demonstrated a significant influence as a predictor as well (Nov *p* = 0.022, uPAR *p* = 0.001). The model comprising the benign and tumor group was not statistically significant. The accuracy was found to be 0.64. A comprehensive overview of the analytical parameters is provided in Table 3.

**TABLE 3:** Results of binary logistic regression models for Nov and uPAR in combination and the clinical characteristics age, gender, and smoking status in addition.

	Predictor	*b*	SE	*z*	*p*	OR	CI
Model 1: Nov and uPAR in combination							
Control and tumor group	*χ* ^2^(2) = 94.13 *p* < 0.001, Cox & Snell *R* ^2^ = 0.63; Nagelkerke *R* ^2^ = 0.84 Sensitivity 91.7%, Specificity 91.3%, Accuracy 91.5%						
	Nov	0.302	0.19	1.57	0.12	1.35	[0.93; 1.97]
	uPAR	2.23	0.56	3.99	< 0.001	9.286	[3.11; 27.76]
Control and benign group	*χ* ^2^(2) = 94.52 *p* < 0.001, Cox & Snell *R* ^2^ = 0.66; Nagelkerke *R* ^2^ = 0.88 Sensitivity 90.2%, Specificity 95.7%, Accuracy 93.1%						
	Nov	0.51	0.22	2.29	0.02	1.67	[1.08; 2.58]
	uPAR	1.54	0.48	3.21	0.001	4.67	[1.83; 11.96]
Benign and tumor group	*χ* ^2^(2) = 3.62 *p* = 0.164, Cox & Snell *R* ^2^ = 0.04; Nagelkerke *R* ^2^ = 0.05 Sensitivity 81.3%, Specificity 43.9%, Accuracy 64%						
	Nov	−0.03	0.05	0.59	0.56	0.97	[0.87; 1.08]
	uPAR	−0.13	0.08	1.40	0.16	0.74	[0.74; 1.05]
Model 2: Nov and uPAR with clinical features (age, gender, smoking status)							
Control and tumor group	*χ* ^2^(5) = 118.36, *p* < 0.001 Cox & Snell *R* ^2^ = 0.72; Nagelkerke *R* ^2^ = 0.96 Sensitivity 97.9%, Specificity 95.7%						
	Age	0.27	0.16	1.70	0.09	1.32	[0.96; 1.80]
	Gender	−3.97	2.78	1.42	0.15	0.02	[0.00; 4.45]
	Smoker	0.93	2.83	0.33	0.74	2.52	[0.10; 645.99]
	Nov	0.20	0.48	0.41	0.68	1.22	[0.48; 3.11]
	uPAR	1.22	0.94	2.36	0.02	9.23	[1.46; 58.26]
Control and benign group	*χ* ^2^(5) = 107.25 *p* < 0.001, Cox & Snell *R* ^2^ = 0.71; Nagelkerke *R* ^2^ = 0.95 Sensitivity 97.6%, Specificity 97.8%						
	Age	0.19	0.13	1.54	0.12	1.21	[0.95; 1.56]
	Gender	−4.23	2.93	1.44	0.15	0.02	[0.00; 4.53]
	Smoker	−6.89	4.58	1.50	0.13	0.001	[0.00; 8.11]
	Nov	0.50	0.35	1.42	0.15	1.65	[0.83; 3.26]
	uPAR	4.38	2.35	1.86	0.06	80.00	[0.80; 8049.33]
Benign and tumor group	*χ* ^2^(5) = 40.10 *p* < 0.001, Cox & Snell *R* ^2^ = 0.36; Nagelkerke *R* ^2^ = 0.49 Sensitivity 83.3%, Specificity 68.3%						
	Age	0.12	0.03	3.75	< 0.001	1.12	[1.06; 1.19]
	Gender	−1.75	0.69	2.52	0.01	0.17	[0.05; 0.68]
	Smoker	0.52	0.59	0.88	0.38	1.68	[0.53; 5.32]
	Nov	−0.11	0.07	1.64	0.10	0.90	[0.79; 1.02]
	uPAR	−0.07	0.11	0.66	0.51	0.01	[0.75; 1.16]

*Note:* In the analysis, gender was replaced by 0 = male, 1 = female, and smoking status by 0 = non‐smoker, 1 = smoker.

Abbreviations: *b* = regression coefficient *b*, CI = confidence interval, OR = odds ratio, *p* = *p*‐value, SE = standard error, *z* = *z*‐value.

Further multivariate logistic regressions were performed to test the influence of age, gender, and smoking in addition to Nov and uPAR. The logistic regression analysis of the control and tumor groups revealed that the model as a whole was statistically significant. In this instance, uPAR was also identified as a significant predictor of diagnosis. Age, gender, smoking status, and Nov were found to have no significant influence. The model incorporating the control and benign groups demonstrated a significant ability to predict benign laryngeal disease. All individual predictors exhibited no significant influence. The multivariate analysis of the benign and tumor groups also showed a significant model, with age and gender identified as having a significant influence on diagnosis. Further details on these analyses can be found in Table 3.

## 4 Discussion

LSCC represents the third most prevalent form of HNSCC in Germany [2]. The most common risk factors for HNSCC include advanced age, male gender, smoking, alcohol consumption, and viral diseases [2, 3]. The tumor stage is also a key determinant of the course of the disease. A more detailed look at LSCC reveals that the early detection of malignant neoplasms is of great importance in terms of overall survival. Chen et al. [45] demonstrate that a T1 tumor has a significantly higher 3‐year survival rate of over 7% compared to a T2 tumor. This emphasizes the crucial importance of early detection. Serum biomarkers become increasingly important in this field due to the non‐invasive nature of sample collection, offering numerous advantages over conventional methods, such as tissue biopsies and laparoscopic explorations. Our previous study demonstrated that exosomal insulin‐like growth factor binding protein 7 (IGFBP7) and annexin A1 (ANXA1) are promising biomarkers for early detection of LSCC [8]. The objective of the present study was to investigate additional suitable non‐invasive biomarkers in greater detail. In a novel approach, Nov and uPAR were examined in serum samples from LSCC patients. Furthermore, this study was conducted in a prospective manner, with serum levels monitored post‐surgery and compared with those of patients with benign tissue changes for the first time.

### 4.1 Serum Findings of Nov and uPAR Confirm Previous Multiplex Proteomic Array Results

The results of the immunohistochemical tissue analysis and serum examinations of LSCC patients compared to the control group demonstrate a clear overexpression of the biomarkers Nov and uPAR. Additionally, the results of the ROC curves demonstrated promising values in relation to diagnostic test quality, particularly for uPAR.

The present study observed the overexpression of Nov in the serum of LSCC patients for the first time. Except for the study performed by Schuster et al. [8], no further research has been conducted on the expression of Nov in LSCC patients. However, previous literature has already confirmed an association between upregulated Nov expression in tumor tissue and severe invasion, poor prognosis, and overall survival in other tumor types, including prostate carcinoma, colorectal carcinoma, triple‐negative breast tumors, and melanoma [13, 46, 47, 48]. The research methods included immunohistochemical tissue analysis, gene expression analysis, and experiments with different cell lines. The effects of Nov on cell adhesion, migration, survival, and angiogenesis are primarily mediated by the binding of extracellular matrix proteins, including integrins [12, 13]. Integrins promote the synthesis of mitogenic growth factors in the cell nucleus and can induce cell movement via chemotaxis [12]. Overexpression in tumor cells appears to favor these mechanisms.

Dankner et al. [46] have demonstrated that Nov plays a role in osteolytic bone metastases in prostate tumors. An overexpression is also associated with increased tumor aggressiveness, lower overall survival, and a higher relapse rate [46]. While our pilot results do not yet provide information on survival and recurrence rates, the overexpression of Nov in prostate cell cultures is congruent with our results in serum from LSCC patients. Son et al. [13] observed overexpression of Nov in triple‐negative breast cancer. A knockout of Nov resulted in a reduction in metastasis and tumor growth [13]. High Nov expression was associated with poorer survival and epithelial‐mesenchymal transition (EMT) [13]. These findings align with our observations of overexpression in LSCC patients. Additionally, Ueda et al. [47] observed comparable results in colorectal cancer. The overexpression of Nov was associated with increased invasiveness, a poorer prognosis, and a reduced survival rate. Vallacchi et al. [48] demonstrated that Nov modulates cell adhesion in melanoma cells through integrins, thereby enhancing their invasive potential. While our results also confirm the overexpression of Nov in serum and tissue in LSCC patients, an association with certain histopathological factors such as invasiveness, lymph node involvement, metastasis, recurrence, and overall survival has yet to be established. This is partly due to the relatively short follow‐up period of 4–8 weeks, which has not yet allowed for the collection of data on recurrence and overall survival. Additionally, the current sample size remains insufficient to enable the meaningful evaluation of certain histopathological criteria. Consequently, the study will be continued.

uPAR interacts with uPA to regulate plasmin production, which facilitates the remodeling of the extracellular matrix and activates matrix metalloproteases. This, in turn, promotes cell migration and the release of angiogenic factors [23, 25, 26]. Additionally, uPAR functions through intracellular signaling pathways, promoting cell proliferation [27]. It appears that overexpression of uPAR serves to enhance these mechanisms. Alongside this, there are already some studies published that demonstrate an overexpression of the biomarker or its ligand uPA in tumor tissue of HNSCC and other locally close squamous cell carcinomas [40, 41, 49, 50, 51, 52]. The results of these studies are also largely based on immunohistochemical analysis of tissue sections and cell culture experiments. The overexpression of uPAR in HNSCC tumor patients, including LSCC, compared to non‐diseased patients has only been detected twice in serum [40, 41]. Risor et al. [41] demonstrated an overexpression of uPAR in the serum of HNSCC patients, five of whom had LSCC. Nevertheless, it was not feasible to anticipate the progression of the disease prior to the initiation of therapy [41]. The soluble form of the urokinase receptor (suPAR) was examined by Schmidt and Hoppe [40]. The study found no correlation between T‐status, metastasis, recurrence, and degree of differentiation [40]. However, due to the small group sizes, these results most probably require further testing in larger samples. In our study, T‐status and uPAR level were found to correlate. Higher serum levels were associated with a higher TNM classification. However, our results so far also come from a small sample and require further verification in the ongoing study.

Parolini et al. [49] demonstrated that uPA is overexpressed in LSCC tissue. One of the principal findings of the study was that uPA expression increased during the transformation of normal laryngeal tissue into a laryngeal tumor [49]. This finding is consistent with our observations on uPAR. Nevertheless, the findings require further validation with a larger sample size. Furthermore, elevated uPA levels were observed in patients with lymph node involvement [49]. This is consistent with the hypothesis that the uPA/uPAR system favors invasiveness and metastasis. This aligns with our findings that uPAR is more elevated in advanced tumors. Conversely, Parolini et al. [49] observed elevated uPA levels primarily in well‐differentiated tumors. However, the authors attributed this to the staining, which cannot distinguish between inactive and active uPAR [49]. Therefore, this finding may not be valid.

### 4.2 Both Benign and Malignant Laryngeal Lesions Result in Elevated Serum Levels of Nov and uPAR Compared to Controls

Compared to controls, significantly increased serum levels of Nov and uPAR were found in both tumor patients and patients with benign laryngeal diseases. The overexpression of the biomarkers in benign laryngeal lesions was particularly surprising. This is probably due to the diseases included in the benign group. Leukoplakia, vocal fold polyps, irritative fibromas, and contact granulomas are associated with local inflammation. Studies have demonstrated that Nov correlates closely with the inflammatory markers IL‐6 and CRP [20, 53]. In addition, the biomarker is also overexpressed in chronic inflammatory processes such as type 2 diabetes mellitus and rheumatoid arthritis and correlates with the inflammatory activity [20, 53]. uPAR is also associated with inflammatory diseases. Sater et al. [54] demonstrated a positive correlation between IL‐6 and uPAR levels. Furthermore, chronic inflammatory bowel diseases exhibit elevated serum levels of uPAR [55]. Consequently, the overexpression of the biomarkers under investigation in patients with benign laryngeal lesions is likely attributable to the inflammatory processes associated with the clinical conditions included. It would be beneficial to conduct further research into the measurement of serum levels of biomarkers in non‐inflammatory benign laryngeal diseases. Furthermore, the biomarkers may be employed for diagnostic purposes in the context of benign lesions as well.

### 4.3 The Levels of Nov and uPAR Remain Elevated Until the First Follow‐Up Time Point in Tumor Patients

In contrast to our initial expectations, the investigated biomarkers did not exhibit a decline in the prospective follow‐up period after the surgical intervention in the tumor patients, despite the removal of the malignant lesions. In the study conducted by Schmidt and Hoppe [40], suPAR levels demonstrated a decline in 8 out of 11 patients following tumor resection, while only three exhibited increasing levels. For this reason, a decrease of the marker was in alignment with our expectations for patients with tumors. One potential explanation for our observation is that these patients frequently undergo additional therapeutic interventions, such as chemotherapy and radiotherapy. This assumption is corroborated by the findings of Risor et al. [41], which demonstrated an elevation in serum uPAR levels in HNSCC patients, including five LSCC cases that underwent radiotherapy, at the 2‐month follow‐up. Consequently, a follow‐up period of 4–8 weeks for tumor patients seems insufficient for the generation of meaningful results. Consistent with this, there is a decrease in both biomarkers in the group of benign laryngeal lesions that are not followed by further therapies.

Strojan et al. [52] examined the expression of uPA in HNSCC tumors. The findings indicated that uPA is more highly expressed in normal laryngeal tissue than in other head and neck tissues [52]. Consequently, the rise in LSCC tumors was also less pronounced in comparison to normal tissue [52]. Furthermore, the elevation in uPA was observed to be less pronounced in recurrent tumors in comparison to primary tumors [52]. The marker remained at a significant level in sera taken before and a median of 54 days after surgery [52]. However, when only the postoperative sera obtained at least 30 days after surgery were considered, a slight but statistically significant increase was observed [52]. This highlights the need for a longer follow‐up period and the observation of uPAR serum levels in recurrences.

A correlation between clinical or histopathological prognostic features was not demonstrated by Strojan et al. [52]. However, we identified a correlation between uPAR level and TNM classification. This may be attributed to the more homogeneous tumor group, which comprises only laryngeal tumors and not all HNSCC.

It is crucial to acknowledge that the findings of our study thus far represent preliminary data from the initial follow‐up. Furthermore, it is necessary to include a larger number of patients in order to obtain significant results. The ongoing study is designed to address the limitations of the pilot data by conducting a longer follow‐up and increasing the size of the participating cohort. The precise pathophysiological mechanisms of Nov and uPAR remain largely unknown and require further investigation to ascertain their suitability as biomarkers. Furthermore, the inclusion of additional markers in the future will facilitate the optimization of therapy monitoring for LSCC and enhance its resilience to interference. Additionally, it will enable a more accurate prediction of recurrence.

### 4.4 Control and Laryngeal Lesions Can Be Well Differentiated by Combining Nov and uPAR

Following the promising diagnostic test quality values yielded by the ROC curves, particularly for uPAR, a binary logistic analysis was conducted to ascertain the predictive capacity of both markers in combination. The results showed that the markers have a high test accuracy for the models control and tumor as well as control and benign group. Furthermore, uPAR has been identified as a potential predictive marker for diagnosis. This is consistent with previous findings in the literature. The combination of different biomarkers has been shown in previous studies to enhance the robustness of the test to confounding factors. Consequently, we will assess this behavior again with a larger number of patients and a broader range of variables, such as prior illnesses and medications, in the next phase of the study. The analysis of the benign and malignant groups did not yield significant results yet and exhibited low accuracy. Neither Nov nor uPAR demonstrated sufficient predictive capacity for diagnosis in this model. Given low *R* ^2^ values for this model, it appeared that a transfer to the total population was also less feasible. As the study is still ongoing, we intend to test this model again in order to achieve a higher level of transferability of the results and a greater degree of reliability in the assessment of these groups. Furthermore, a deeper understanding of the pathomechanisms of Nov and uPAR in relation to benign diseases is necessary. The behavior of the biomarkers in other benign laryngeal diseases of non‐inflammatory origin would also be of interest.

The analysis of the influence of age, gender, and smoking status, in addition to the two biomarkers, demonstrated significant results for all models in predicting the diagnosis. The sensitivity and specificity for the models comparing the control and tumor groups, as well as the control and benign groups, exceeded 95%. Furthermore, uPAR was identified as a significant predictor when analyzing control and LSCC patients. These findings support the potential of uPAR as a biomarker for the diagnosis of laryngeal carcinoma. Nevertheless, the differentiation between benign and malignant remains challenging, as the influence of Nov and uPAR on this prediction was found to be minor and thus requires verification in a larger sample size over the course of the study. The regression analysis for this group constellation was significant, but the test quality was inferior to that observed in the other models. Age and gender were identified as influential predictors in this model. The fact that the benign group is somewhat younger and contains more female subjects may be a contributing factor to these results. Consequently, we will re‐evaluate this analysis as the study progresses.

### 4.5 Relevance to Other Tumor Entities

The present study has thus far demonstrated that the two non‐invasively obtained biomarkers can provide diagnostic and therapeutic indications of the presence and course of laryngeal carcinomas. This represents a significant advantage over other potential LSCC biomarkers, such as Ki‐67, EGFR, and TGF‐β, which are obtained through invasive biopsy procedures [56, 57]. Additionally, Nov and uPAR have been demonstrated to interfere with crucial biological processes, including proliferation and metastasis. These central tumor mechanisms are relevant in many tumor entities. Consequently, these biomarkers are likely to play an important role in many other tumors as well. In line with this, increased Nov and uPAR expressions have already been measured in many different tumors. The results for uPAR in LSCC are comparable to those observed in studies involving HNSCC patients. However, there are currently no studies on HNSCC patients for Nov. Given the similarities in tissue and localization in HNSCC, we anticipate that our results for both markers will be transferable to this group. However, it should be noted that the HNSCC group is significantly more heterogeneous than the LSCC tumor group. Consequently, the results for HNSCC tumors may exhibit greater diversity.

But further knowledge is required to elucidate the pathomechanisms of Nov and uPAR in greater detail and to confirm their suitability as biomarkers. Nevertheless, they may potentially influence clinical decision‐making. In the context of suspected LSCC, uPAR may potentially serve as a biomarker to distinguish between benign and malignant changes in the larynx, particularly when a blood sample is obtained prior to a panendoscopy. Moreover, both biomarkers could be employed in the follow‐up period to facilitate the earlier recognition of recurrence. In instances where clinical findings suggest the possibility of recurrence, the biomarkers may initially be employed to ascertain the likelihood of renewed tumor growth. Subsequently, the necessity for an invasive panendoscopy with sampling can be evaluated. It is our intention to continue this study in order to provide further insights into these aspects.

To gain further insight into the precise pathomechanisms of the biomarkers, it is recommended that in vitro experiments be conducted, in which the steps of the uPAR and Nov signaling pathways are disrupted and the impact on cell processes is then evaluated. Subsequently, these findings could be validated in vivo in LSCC cell lines.

A larger number of test subjects, a longer follow‐up period, and further investigation of recurrence patterns, as planned for in this study, would provide more precise information about the behavior of the biomarkers. The combination of Nov and uPAR with other biomarkers could enhance the specificity and sensitivity. In the study conducted by Risor et al. [41], uPAR PET/CT was also found to be a promising non‐invasive approach for forecasting the progression of disease. This technique may be employed in conjunction with serum biomarkers. Furthermore, blinded studies should be conducted in which patients are allocated to groups based on the measured serum concentrations of the two biomarkers. The correctness of the allocation to the control, benign, and malignant group is then checked. The primary, blinded allocation may be based on the cut‐off values of the ROC curves.

### 4.6 Limitations

The study results to date are subject to certain limitations. These limitations are caused, among other things, by a small group size and the use of pilot results, which only describe a minimal follow‐up period. Both concerns should be addressed by the continuation of the study.

Furthermore, only patients from one ENT center in Germany were included in this pilot study, all of whom were of Caucasian ethnicity. In order to facilitate the generalizability of the results, it would be beneficial to include a cohort from multiple centers in different countries. The inclusion and exclusion criteria resulted in the inclusion of only certain diagnoses and previous diagnoses, which also introduced confounding factors and participation bias. The comparison of pre‐ and postoperative serum markers only included patients who underwent surgery, which may lead to a study examination bias. Moreover, a loss to follow‐up bias may be present, as the control group was not included in the follow‐up. However, as we assume constant concentrations in this group, this should be minimal. In general, efforts were made to minimize the impact of confounding factors and potential biases. Some factors, such as examination bias, were difficult to address. This was due to the inability to compare biomarker levels after tumor reduction or removal without intervention. Due to the limited size of the study group, the observed effect is merely indicative. It is imperative that a larger number of test subjects be included in the study, as well as that further comparisons be made with benign laryngeal diseases. These are the primary objectives of the ongoing study.

## 5 Conclusion

Nov and uPAR appear to be promising biomarkers for differentiating between healthy individuals and patients with laryngeal lesions. In this prospective study, the inclusion of benign lesions was a novel approach. Furthermore, the analysis revealed that uPAR exhibited the capacity to differentiate between T1 and benign tumors, as well as the control group. The BEAL study is ongoing to generate more meaningful results with a larger, more diverse study cohort and a longer postoperative follow‐up, including data on recurrence and survival rates. The findings obtained in our study so far also allow the recommendation to investigate further benign laryngeal lesions without inflammatory processes in the follow‐up.

## Author Contributions

Conceptualization: S.K.M., O.W., M.H., B.F. Methodology: O.W., A.‐C.W. Software: O.W., A.‐C.W. Validation: S.K.M., O.W. Formal analysis: A.‐C.W. Investigation: A.‐C.W. Resources: S.K.M., J.S., A.‐C.W., A.A. Data curation: A.‐C.W. Writing – original draft: A.‐C.W. Writing – review and editing: S.K.M., O.W., A.‐C.W., M.H., B.F., A.A. Visualization: O.W., A.‐C.W. Supervision: S.K.M., O.W. Project administration: S.K.M.

## Disclosure

The present work was performed in partial fulfillment of the requirements for obtaining the degree Dr. med.

## Ethics Statement

No. 451_20 B by the ethics committee of the Friedrich‐Alexander‐University Erlangen‐Nuremberg.

## Consent

Informed consent was obtained from all subjects involved in the study.

## Conflicts of Interest

The authors declare no conflicts of interest.

## Data Availability

The data that support the findings of this study are available from the corresponding author, upon reasonable request.
